# Distinguishing Ichthyoses by Protein Profiling

**DOI:** 10.1371/journal.pone.0075355

**Published:** 2013-10-09

**Authors:** Robert H. Rice, Katie M. Bradshaw, Blythe P. Durbin-Johnson, David M. Rocke, Richard A. Eigenheer, Brett S. Phinney, Matthias Schmuth, Robert Gruber

**Affiliations:** 1 Department of Environmental Toxicology and Forensic Science Graduate Program, University of California Davis, Davis, California, United States of America; 2 Division of Biostatistics, Department of Public Health Sciences, Clinical and Translational Science Center Biostatistics Core, University of California Davis, Davis, California, United States of America; 3 Division of Biostatistics, Department of Public Health Sciences, Clinical and Translational Science Center Biostatistics Core and Department of Biomedical Engineering, University of California Davis, Davis, California, United States of America; 4 Proteomics Core Facility, University of California Davis, Davis, California, United States of America; 5 Department of Dermatology and Venereology, Innsbruck Medical University, Innsbruck, Austria; University of Maryland School of Medicine, United States of America

## Abstract

To explore the usefulness of protein profiling for characterization of ichthyoses, we here determined the profile of human epidermal stratum corneum by shotgun proteomics. Samples were analyzed after collection on tape circles from six anatomic sites (forearm, palm, lower leg, forehead, abdomen, upper back), demonstrating site-specific differences in profiles. Additional samples were collected from the forearms of subjects with ichthyosis vulgaris (filaggrin (*FLG*) deficiency), recessive X-linked ichthyosis (steroid sulfatase (*STS*) deficiency) and autosomal recessive congenital ichthyosis type lamellar ichthyosis (transglutaminase 1 (*TGM1*) deficiency). The ichthyosis protein expression patterns were readily distinguishable from each other and from phenotypically normal epidermis. In general, the degree of departure from normal was lower from ichthyosis vulgaris than from lamellar ichthyosis, parallel to the severity of the phenotype. Analysis of samples from families with ichthyosis vulgaris and concomitant modifying gene mutations (*STS* deficiency, *GJB2 deficiency*) permitted correlation of alterations in protein profile with more complex genetic constellations.

## Introduction

The ichthyoses, or synonymous generalized Mendelian disorders of cornification (MEDOC), comprise a diverse group of scaling disorders of most or all of the skin. The current classification of ichthyoses is predominantly clinically based; i.e., through inspection they can be distinguished by type and distribution of scaling and hyperkeratosis, presence/absence of concomitant erythema, and nail or hair involvement [1]. Family history (mode of inheritance), onset of phenotypic changes and involvement of other organ systems provide additional clues for differential diagnosis [1], [2]. In some, but not all subtypes, sampling of the skin for laboratory procedures involving detergent- and heat-exposure of scale, zymography and immunostaining combined with light and electron microscopy aid in the differential diagnosis.

Since the genetic basis for most types of ichthyosis has been elucidated, this information is now increasingly used for differential diagnosis and nomenclature, e.g., LI(*TGM1*) for lamellar ichthyosis (LI) due to transglutaminase-1 deficiency. Disease causing mutations in MEDOC comprise various genes, mainly involving epidermal barrier and keratinocyte differentiation [1], [2]. In recent years, considerable progress has been made toward a better understanding of their pathogenesis [3], [4]. This includes defects in enzymes responsible for the lipid barrier that prevent abnormal transepidermal water loss, structural proteins that provide a scaffold for the lipid barrier and are involved in keratinocyte homeostasis, cell-cell junction proteins, and other mechanisms [2], [5]. The process of identifying contributing genes, including those involved in lipid processing, continues [6], [7], [8], [9]. Considerable effort has been devoted to understanding genotype to phenotype correlations, including recent work showing deficiency in filaggrin (FLG) as a prime factor in ichthyosis vulgaris (IV) [10], *STS* deletions resulting in altered ultrastructure and gene expression [11], [12], defects in ATP-binding cassette subfamily A12 (ABCA12) responsible for harlequin ichthyosis [13], [14], and mutations in transglutaminase 1 (*TGM1*) causing approximately one-third of autosomal recessive congenital ichthyoses and the majority of the LI subtype [15], [16].

The ichthyosis phenotype is a reflection of the genotype, environmental influences and the effort of the epidermal cellular machinery to restore disturbed differentiation and barrier function. In some instances, the severity is influenced by the coincidence of more than a single predisposing gene defect. For example, X-linked ichthyosis (XLI) can be exacerbated by a concomitant *FLG* mutation acting as a modifier [17]; however, other yet unknown genes could also modify the phenotype [18].

Thus, a need exists to assess epidermal expression levels of genes that may influence cellular function. Despite increasing availability of molecular analysis to identify underlying gene mutations in the ichthyoses, corresponding alterations in protein expression are largely unknown, although they can conveniently be analyzed in non-invasively sampled scale. In addition, monitoring of novel therapies in a non-invasive way by measuring stratum corneum (SC) protein patterns is a valuable goal. Recent findings illustrate the usefulness of proteomic analysis of hair shaft corneocytes for observing manifestations of genetic variation [19]. Applications to samples derived from normal and LI epidermis have also been demonstrated [20].

Present work first characterizes epidermal SC proteins obtained by tape stripping at several anatomic sites to demonstrate the feasibility of performing protein profiling and the importance of comparing normal and afflicted epidermis at the same site. This approach provided a foundation for analyzing cases of LI and IV with and without concomitant gene defects, AD and XLI. The results substantiated our hypotheses that significant differences in protein profiles between the major ichthyosis subtypes could be discerned, and that the profiles could reveal individuals with both a main causal underlying gene defect and concomitant modifier genes.

## Materials and Methods

### Study Participants

The 21 individuals studied include 8 patients with IV (2 compound heterozygous and 6 heterozygous *FLG* mutations), one patient with atopic dermatitis (AD, heterozygous for *FLG*), 2 patients with LI (*TGM1* mutations), 2 patients with XLI (*STS* deletions), and one female asymptomatic *STS* deletion-carrier. Clinical diagnoses were made by two experienced dermatologists. As controls, we included 7 healthy individuals lacking scaling or any other inflammatory skin symptoms.

### Ethics Statement

The subjects in this study (Table 1) were recruited with written informed consent. From the minor male patient additional written informed consent was obtained from his parents. The study was approved by the Institutional Review Boards of the Innsbruck Medical University, Innsbruck, and the University of California, Davis, and complied with the Declaration of Helsinki Principles.

**Table 1:** Overview of analyzed samples.

Subject	Sex	Age (yrs)	Diagnosis	Mutation genotype
**Normal controls for stratum corneum depth and anatomical site analysis**				
Ctl1	F	23	Normal skin	FLG wt/wt
Ctl2	M	29	Normal skin	FLG wt/wt
Ctl3	M	65	Normal skin	FLG wt/wt
Ctl4	F	28	Normal skin	FLG wt/wt
**Subjects with Ichthyosis, atopic dermatitis and normal controls for protein profiling**				
S1	F	28	IV, mild, Hy^a^	FLG 2282del4/wt
S2	M	33	IV, medium, Hy	FLG R501X/2282del4
S3	M	65	IV, severe, Hy	FLG R501X/R2447X
S4 (Cmpd2)	F	34	IV, mild, Hy	FLG R501X/wt; GJB2−/− (p.G11fsX59/p.L90P)
S5	F	33	AD, nonlesional skin, Hy	FLG 2282del4/wt
S6	M	38	LI, severe	TGM1−/− (Pro118Leu); FLG wt/wt
S7	M	24	LI, severe	TGM1−/− (Pro118Leu); FLG wt/wt
Ctl8	F	30	Normal skin	FLG wt/wt
Ctl9	M	30	Normal skin	FLG wt/wt
Ctl10	M	33	Normal skin	FLG wt/wt
**Family I**				
Father	M	60	IV, medium, Hy	FLG 2282del4/wt
Mother	F	57	Normal skin	FLG wt/wt
Daughter	F	29	IV, medium, Hy	FLG 2282del4/wt
**Family II**				
Father	M	60	IV, mild, Hy	FLG 2282del4/wt
Mother	F	58	Carrier XLI	Microdeletion STS on Xp22.32; FLG wt/wt
Daughter	F	30	IV, mild, Hy	FLG 2282del4/wt
Son1 (Cmpd1)	M	19	XLI, medium, Hy	Microdeletion STS on Xp22.32; FLG 2282del4/wt
Son2 (Cmpd1)	M	16	XLI, severe, Hy	Microdeletion STS on Xp22.32; FLG 2282del4/wt

a Hy, palmar hyperlinearity.

### Genotyping

Genomic DNA was extracted from peripheral blood using the GenoM48 automated extractor (Qiagen, Vienna, Austria). Screening for *FLG* mutations was performed as described previously [10]. Genomic microdeletions of the *STS* gene on Xp22.32 were detected by FISH as reported formerly [18], and screening for *TGM1* mutations was performed as reported previously [21].

### Sample Preparation

Samples of SC were collected using 22 mm diameter tape circles from D-Squame Pro Kits (CuDerm Corp, Dallas, TX). For measurements of SC protein with depth, on the forearm 10 consecutive circles were pooled for each of 5 depths. For comparison of anatomic sites on forearm, palm, lower leg, forehead, abdomen and upper back, 5 circles were collected, respectively, starting at the surface and pooled. For subjects of known genotype, 2–5 samples were analyzed each containing 2–8 circles. Tapes were applied to the skin with pressure using a strong circular motion, transferred (adhesive side toward the center) to sterile new plastic or glass tubes and covered with a solution of 2% sodium dodecyl sulfate – 0.1 M sodium phosphate, pH 7.8. The tubes were incubated at room temperature for 1–2 days, during which time the cells eluted from the tapes and accumulated at the bottoms of the tubes. The cells were removed by pipetting, rinsed twice with the sodium dodecyl sulfate-sodium phosphate buffer and resuspended in 0.4 ml of buffer. Protein disulfides were reduced in 25 mM dithioerythritol and then alkylated with 50 mM iodoacetamide. Protein was precipitated by addition of 1 ml of ethanol, rinsed twice with 67% ethanol and once with fresh 0.1 M ammonium bicarbonate. The protein was digested in 0.4 ml of ammonium bicarbonate - 10% acetonitrile by addition of 20 µg of reductively methylated bovine trypsin [22] added at daily intervals. After three days, the digest was clarified by centrifugation, and the supernatant was submitted for mass spectrometric analysis. For immunoblotting, samples eluted from tape circles were electrophoresed on 10% gels, transferred to immobilon membranes and detected using rabbit monoclonal antibodies to KRT6 (EPR1602Y, recognizing KRTs 6A, 6B, 6C), KRT9 (EPR10932) or KRT16 (EP1615Y), all from Abcam (Cambridge, MA), or mouse monoclonal antibody to KRT10 (DE-K10) from Thermo Fisher Scientific (Waltham, MA).

### Mass Spectrometry and Protein Identification

The samples adjusted to approximately equal peptide amounts by A^280^ were acidified with trifluoroacetic acid and loaded onto an Agilent ZORBAX 300SB C_18_ reverse-phase trap cartridge, which was then switched in-line with a Michrom Magic C_18_ AQ 200 µm×150 mm nano-LC column connected to a Thermo-Finnigan LTQ iontrap mass spectrometer through a Michrom Advance Plug and Play nanospray source with CTC Pal autosampler. The nano-LC column was used with a binary solvent gradient; buffer A was composed of 0.1% formic acid and buffer B composed of 100% acetonitrile. The 120 min gradient consisted of the steps 2–35% buffer B for 85 min, 35–80% buffer B for 23 min, hold for 1 min, 80-2% buffer B for 1 min, then hold for 10 min, at a flow rate of 2 µl/min for maximal separation of tryptic peptides. An MS survey scan was obtained for the m/z range 375–1400, and MS/MS spectra were acquired from the 10 most intense ions in the MS scan by subjecting them to automated low energy CID. An isolation mass window of 2 Da was used for the precursor ion selection, and normalized collision energy of 35% was used for the fragmentation. A 2 min duration was used for the dynamic exclusion.

Tandem mass spectra were extracted with Xcalibur version 2.0.7. All MS/MS samples were analyzed using X! Tandem (The GPM, thegpm.org; version TORNADO (2010.01.01.4)). X! Tandem was set up to search a June 6, 2012 Uniprot human complete database (261,004 proteins), appended to an identical but reversed database for calculating false discovery rates, assuming the digestion enzyme was trypsin. X! Tandem was searched with a fragment ion mass tolerance of 0.40 Da and a parent ion tolerance of 1.8 Da. Iodoacetamide derivative of cysteine was specified in X! Tandem as a fixed modification. Deamidation of asparagine and glutamine, oxidation of methionine and tryptophan, sulfone of methionine, tryptophan oxidation to formylkynurenin of tryptophan and acetylation of the N-terminus were specified in X! Tandem as variable modifications. Scaffold (version Scaffold_3.2.0, Proteome Software Inc., Portland, OR) was used to validate MS/MS based peptide and protein identifications. Peptide identifications were accepted if they could be established at greater than 90% probability as specified by the Peptide Prophet algorithm (false discovery rate 0.14%) [23]. Protein identifications were accepted if they could be established at greater than 99% probability and contained at least 2 identified peptides (false discovery rate 1.7%). Protein probabilities were assigned by the Protein Prophet algorithm [24]. Proteins that contained similar peptides and could not be differentiated based on MS/MS analysis alone were grouped to satisfy principles of parsimony. Numbers of assigned spectra were tabulated, and assigned spectra (Table S1) were adjusted for shared peptides [25] using a locally developed script [26].

### Statistical Analysis

Median normalization was conducted separately across each analysis dataset. Thus, the adjusted spectral counts were additively adjusted so that the median spectral counts across peptides were the same for each sample. Adjusted spectral counts were then analyzed using mixed-effects overdispersed Poisson regression models including a random effect for subject and, where applicable, a random effect for family, to account for correlation between samples for the same subject and between subjects from the same family. Models also included a fixed effect for sample depth where applicable; however, results for depth are reported only for analyses for which depth was of specific interest. When more than 2 levels of a factor were compared (such as for site or depth), the Tukey HSD method was used to adjust for multiple testing of all pairwise comparisons (the exception being depth in the analysis of ichthyosis patients, for which p-values were not adjusted). Mixed effects overdispersed Poisson regression modeling was conducted using the glmmPQL function in R, version 2.13.0 (R Development Core Team, 2011).

Proteins that were absent from a preponderance of the samples were deleted from the data prior to analysis. Comparisons among subjects from family I and subjects from family II, respectively, were conducted using fixed effects overdispersed Poisson regression, using the glm function in R, followed by Tukey HSD pairwise comparisons. The data and the code in R to perform the analysis are available from Dr. Durbin-Johnson.

Hierarchical clustering of diagnoses and of family members in each family was performed using the hclust function in the R statistical software environment using the complete linkage method as described in http://nlp.stanford.edu/IR-book/html/htmledition/single-link-and-complete-link-clustering-1.html (Manning et al, 2008). For the purposes of hierarchical clustering, the distance between two diagnoses or subjects was defined as the number of significant pairwise differences from the overdispersed Poisson regression analysis.

All graphs depict the results as mean ± standard deviation after normalization.

## Results

Shotgun mass spectrometric analysis of the samples obtained from D-Squame tape stripping of the forearm of three pilot subjects permitted identification of 81 proteins (Table S1) by the stringent criteria employed. Keratins comprised more than one-quarter of the proteins and accounted for the large majority of peptides matched to the database. Among the keratins identified, the most prominent (in order of spectral count) were KRT2, KRT10, KRT1, KRT9 and KRT5. Prominent among the non-keratins were junctional proteins (DSG1, DSP, JUP, DSC1), transglutaminases (TGM1, TGM3), transglutaminase substrates (KPRP, LOR, FLG) and related proteins (FLG2, HRNR).

### Variation of Protein Profiles with Depth and Location

**Figure 1:**
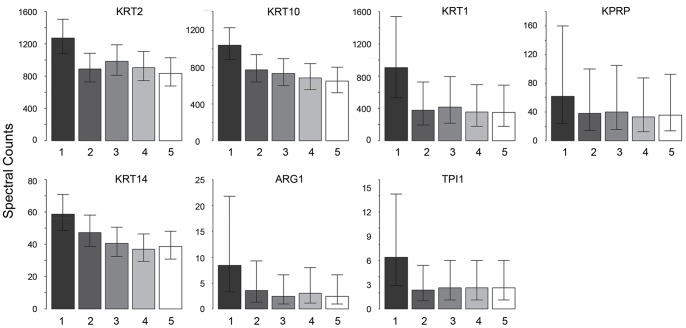
Variation of protein profiles with depth in SC. Relative yields of proteins changing consistently with depth in SC. Each depth level represents 10 pooled tape circles (depth 1 = circles 1–10, depth 2 = circles 11–20, etc.) from three normal subjects.

**Figure 2:**
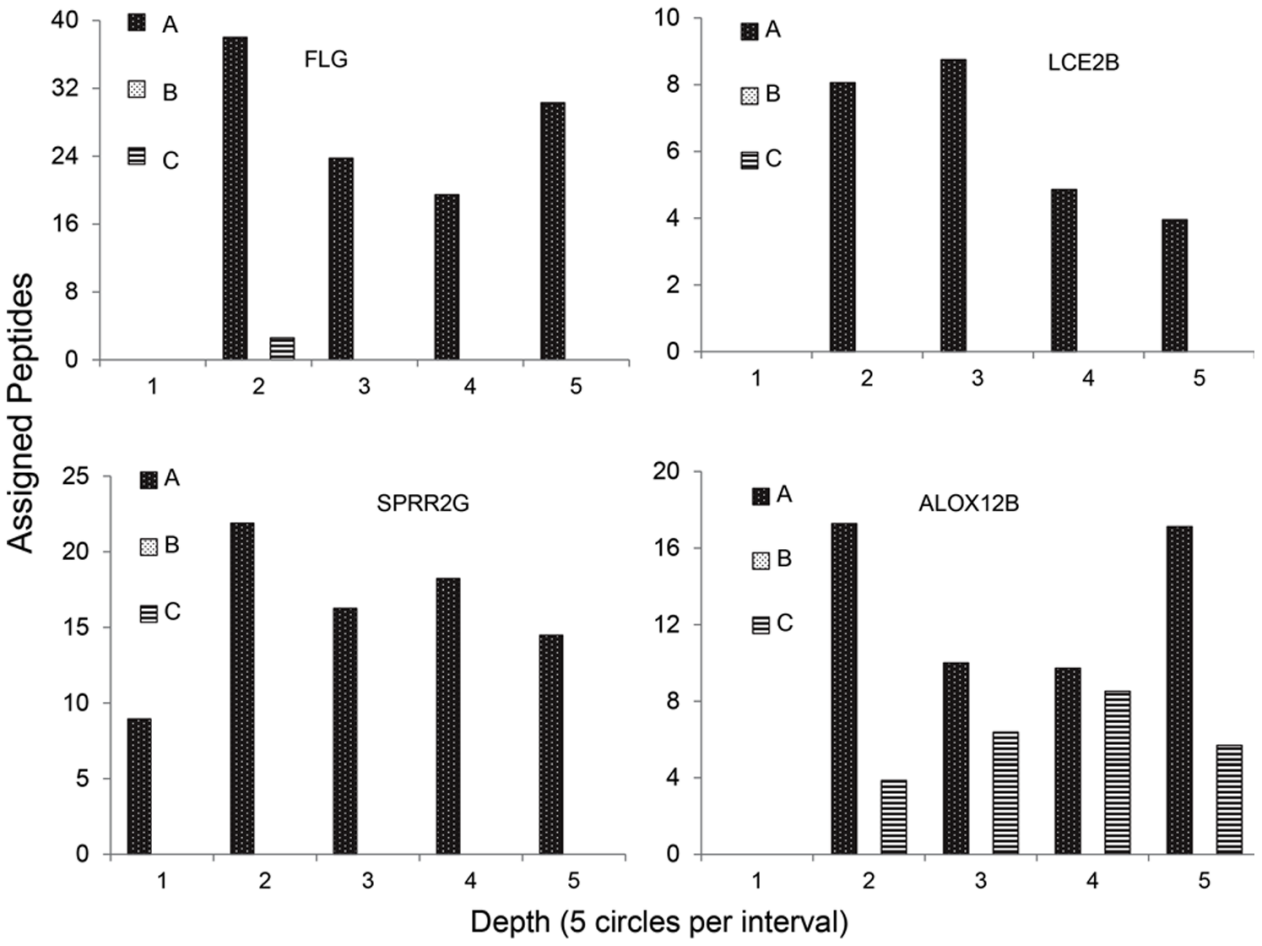
Inter-individual variation of protein profiles. Individual differences in relative yields of proteins from three normal subjects (A (Ctl3), B (Ctl2), C (Ctl1)). Three proteins (FLG, LCE2B, ALOX12B) were not detected in the most superficial sample (1). Two proteins were detected in only two subjects, and two in only one subject.

Statistical analysis was performed of the protein yield according to depth from the surface of the epidermis (Table S2). This analysis revealed that several of the most prominent proteins (KPRP, KRTs 1, 2, 10, 14) and two with fewer spectral counts (ARG1, TPI1) displayed their highest yields at the skin surface (Figure 1). Other less abundant SC proteins showed considerable interindividual variation in their spectral counts, e.g., SPRR2G and LCE2B were detected in only 1 of 3 subjects (Figure 2).

**Figure 3:**
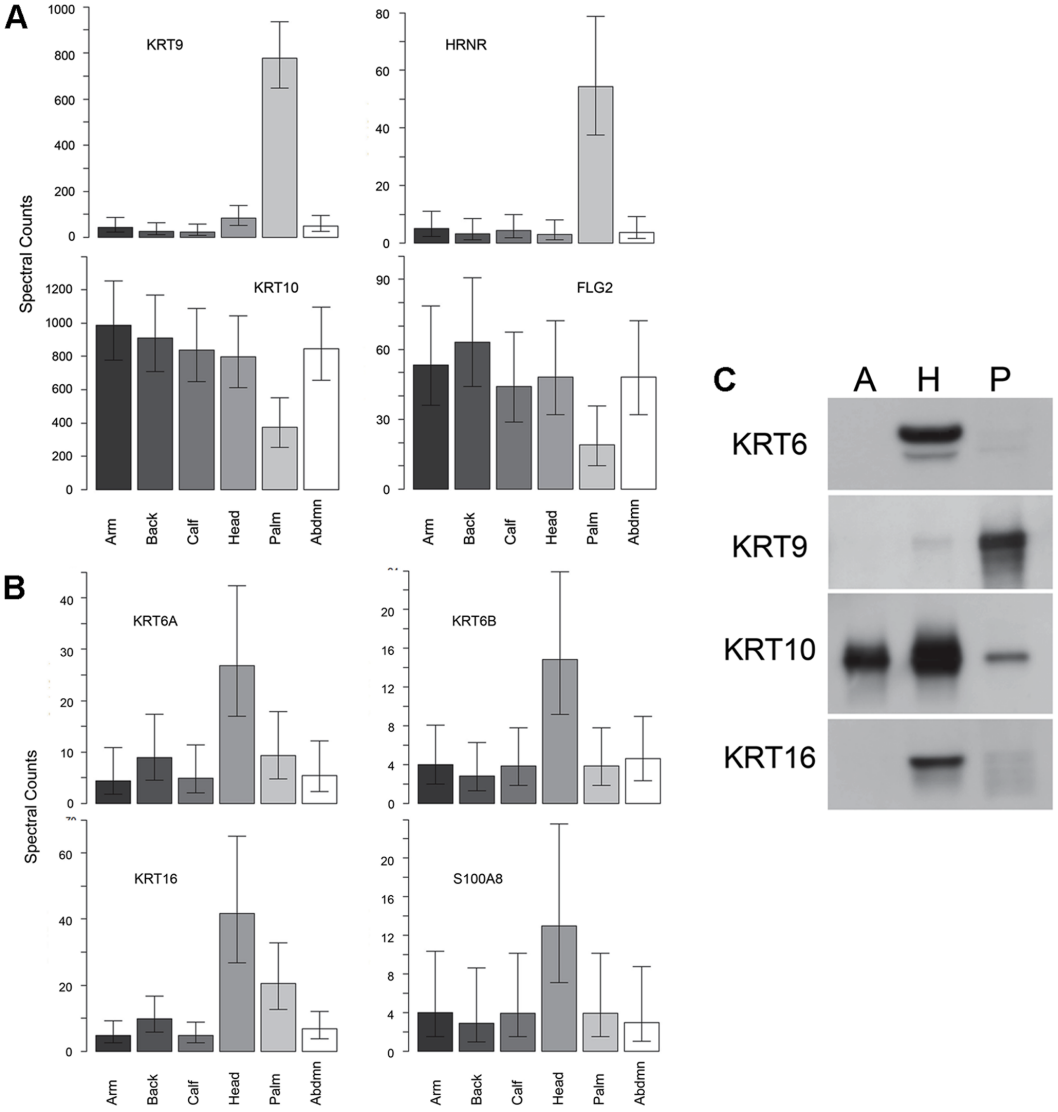
Variation of protein profiles with body site. Differences in epidermal protein profiles among anatomic sites in four normal subjects (Ctls 1–4). Shown are 4 proteins distinguishing the profile of (**A**) palm and (**B**) (fore)head from the other sites. (**C**) Comparisons of relative levels of KRTs 6, 9, 10 and 16 in arm (A), forehead (H) and palm (P) by immunoblotting in samples obtained with tape circles from a normal subject.

In a second set of samples, the proteins identified in the outer layers of the epidermis from the (fore)arm were compared to those obtained in the outer layers at five other locations, i.e., palm, lower leg, (fore)head, abdomen and upper back. All told, 86 proteins were identified, of which 79 were prevalent enough for statistical analysis (Table S3). Those with the most distinctive distributions were KRT9 and HRNR (most prevalent in palm, Figure 3A) and KRT6A, KRT6B, KRT16 and S100A8 (most prevalent in forehead, Figure 3B). As seen in the table of 2-way comparisons among the proteins showing significant differences in spectral counts and the hierarchical distribution derived from it (Figure S1A), arm, back and calf exhibited the fewest differences among them, palm and abdomen were similar, and (fore)head exhibited many differences from the others. The differences in levels of KRTs 6, 9, 10 and 16 were readily demonstrable by immunoblotting proteins extracted from SC samples provided by a normal subject (Figure 3C).

### Distinguishing Ichthyosis Subtypes by Protein Profiles

**Figure 4:**
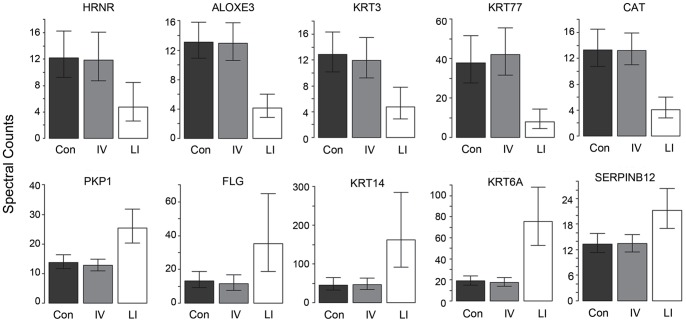
Different protein profiles in ichthyoses and controls. Proteins illustrating differences in profiles of lamellar ichthyosis (LI) versus ichthyosis vulgaris (IV) and controls (Con). The top row shows proteins that are lower in LI than in IV and control samples, while the bottom row shows proteins that are elevated in LI.

The (fore)arm samples from 4 additional normal subjects, two cases of LI (*TGM1*−/−) and 8 cases of IV patients were analyzed for proteomic profile (Table S4). Along with the 3 previously collected arm samples, these were then subjected to statistical testing to find whether normal, LI and IV were distinguishable by proteomic profiling (Table S5). Hierarchical clustering of the pairwise differences gave clear separation of the 3 groups as shown in Figure S1B. Of the 65 proteins compared in this analysis, 47 showed significant differences among these 3 groups, with 9 distinguishing IV from the normal control category, and 38 distinguishing LI and IV. Among the latter were numerous keratins as well as junctional and cytoplasmic proteins. As illustrated by the sample of 10 of these proteins in Figure 4, equal numbers in each category were increased or decreased.

**Figure 5:**
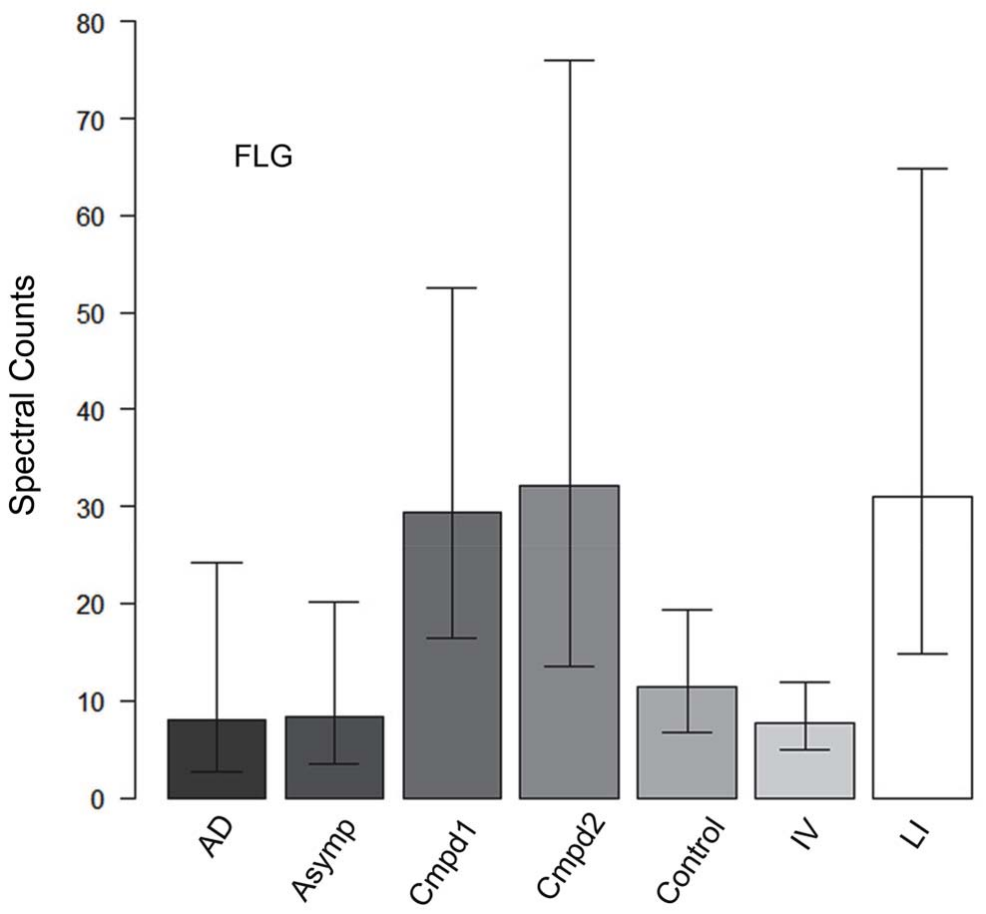
Different levels of filaggrin (FLG) in genodermatoses and controls. Comparison of FLG levels among the samples with various diagnoses. AD, atopic dermatitis (nonlesional skin); Asymp, asymptomatic carrier of *STS* microdeletion; Cmpd1, *FLG* defect with *STS* microdeletion; Cmpd 2, heterozygous *FLG* defect with concomitant *GJB2* mutation.

Whether epidermal samples from participants with other diagnoses could be distinguished by pairwise protein comparisons was tested (Table S6). As shown in Figure S1C, inclusion of a sample of AD (nonlesional skin) was not distinguishable from the control samples; however, one from an asymptomatic female with an *STS* microdeletion was distinguishable. Moreover, samples from individuals heterozygous for a *FLG* mutation and a concomitant defect, either of connexin *GJB2* (Compound 2) or an *STS* microdeletion (Compound 1, two males) were distinguishable from the others. One striking difference among the samples was the high level of FLG from the subjects with LI and the subjects with compound defects compared to the others (Figure 5).

### Distinguishing Family Members by Protein Profiles

**Figure 6:**
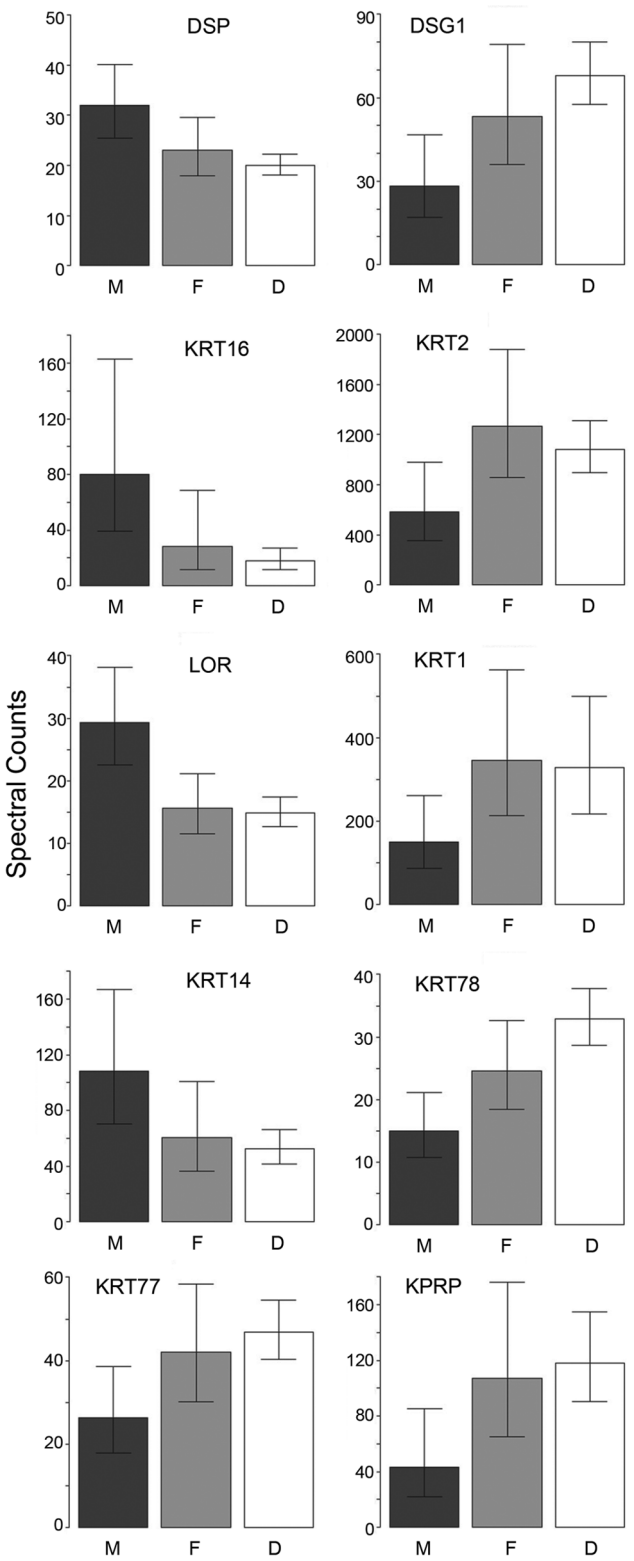
Intra-familial differences in protein profiles. Examples of proteins differing in family I between normal subject and two family members with IV. Illustrated are 4 proteins in which the levels in the normal subject (M) are higher than the spouse (F) and offspring (D), and 6 proteins where the levels are lower.

Protein profile differences were then examined in two families containing individuals that are heterozygous for *FLG* mutations. In family I, samples from the mother with normal phenotype and father and daughter with an IV phenotype exhibited 29 significant differences in pairwise comparisons among the 65 proteins analyzed (Table S7), permitting the hierarchical distribution illustrated in Figure S1D. Among these, and illustrated in Figure 6, are 10 proteins that consistently differed between the normal parent and the two IV family members.

**Figure 7:**
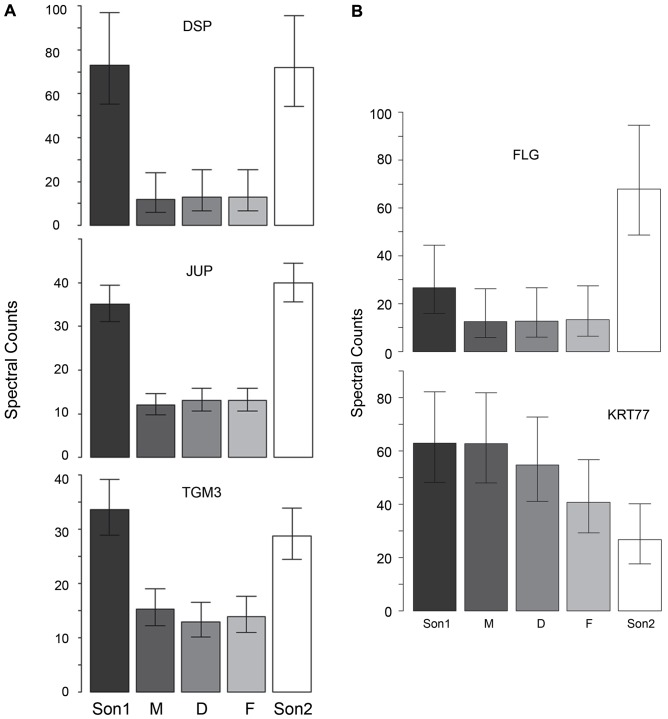
Intra-familial differences in protein profiles. Proteins are significantly different between the samples from family II subjects with heterozygous *FLG* mutation and those with concomitant *FLG* mutation and *STS* microdeletion. Shown are samples from the father (F), mother (M), daughter (D) and two sons (Son1, Son2). (**A)** Proteins DSP, JUP, TGM3 are elevated in Son1 and Son2. (**B)** Comparison of FLG and KRT77 among family members, with striking differences between son1 and son2.

Family II consisted of a phenotypically normal mother with a carrier status for an *STS* microdeletion. The father and daughter presented with IV and a heterozygous *FLG* mutation. Two sons exhibited variable phenotypic severity of XLI with palmar hyperlinearity, where both displayed concurrent heterozygous *FLG* mutations and concomitant *STS* microdeletions [18]. Of the 65 proteins analyzed, 34 exhibited significant pairwise differences among the 5 family members (Table S8). As is evident in the hierarchical clustering of these differences (Figure S1E), the two sons displayed the most protein differences from the other family members. Of these, 3 (DSP, JUP, TGM3) were consistently detected at higher levels in comparison with the two other family members exhibiting the IV phenotype (Figure 7). The two sons differed phenotypically in severity. Samples from the one with the more severe phenotype exhibited 3 fold the level of FLG and one third the level of KRT77 of the one with the less severe phenotype (Figure 7).

## Discussion

Present results show that shotgun proteomics can identify well over 50 proteins in human SC. Tape stripping of various anatomic sites (forearm, palm, lower leg, forehead, abdomen, upper back) revealed differences in the relative yields of proteins, where the pattern from palm exhibited striking differences from the others, chiefly in KRT9 and HRNR. Most of the proteins did not change much in yield from exterior to interior of the SC, indicating relative insensitivity to endogenous proteolysis in the corneocytes. Several proteins exhibited maximal levels in the superficial layers, perhaps due to their continued incorporation into cross-linked material as the squames transit outward [27]. Some proteins displayed marked differences in yield among individuals not attributable to a gradient with depth.

Comparisons among the various ichthyosis samples analyzed showed clear differences. In global comparisons, these were few in number between normal and IV categories, but marked comparing the LI cases to others. Inspection of the data revealed more alterations of keratins, junctional and other proteins as the degree of phenotypic severity increased, parallel to the degree of barrier function deficiency. Now that comparing protein levels among samples from lesional epidermis is feasible, finding whether such measurements have diagnostic utility is possible. While the degree of departure from the normal protein profile is likely related to the phenotypic severity, whether the pattern reflects the basic defect or is generic among a variety of basic defects remains to be established. Analysis of patterns from a larger sampling of subjects of characterized genotype is anticipated to resolve this uncertainty. The present results do not include a systematic comparison between SC shotgun proteomics and known changes in cell structure in the ichthyoses studied, i.e., alterations in intermediate filament and cornified envelope morphology. Nevertheless, the compensatory increase in FLG expression that we observed in subjects with LI (*TGM1*−/−) appears to correlate with the relatively mild morphologic attenuation of cornified envelopes reported in LI [28], [29].

Evidence for variation in baseline levels of certain proteins influencing the phenotype might be difficult to resolve over the homeostatic response. However, analysis of families, which minimizes wide population variance, offers the possibility to make more direct inter-individual comparisons. For example, in one of the families analyzed (Table 1, family II), the influence of a concomitant *STS* microdeletion was clearly seen. A notable feature of the samples from the two brothers, both showing the heterozygous *FLG* mutation 2282del4 and an *STS* microdeletion, was a high expression level of FLG. This was also evident in samples from a subject with IV, showing the same *FLG* mutation status but a concomitant *GJB2* mutation (Tables 1, S4) and the two patients with LI (Tables 1, S6 and S7), suggesting compensatory mechanisms.

The present shotgun approach provides a good estimate of differences among the most prevalent proteins. It would readily detect loss of a prominent protein due to a homozygous genomic deletion or premature termination codon. By contrast, loss of FLG (unlike FLG2) would not so easily be noticed at its low level of detection in present work, but the several fold increase in sensitivity in newer instruments becoming available likely will alleviate this difficulty. Point mutations or changes in specific regions in proteins of interest (e.g., by alternate splicing or post-translational modification) would be challenging to detect in this way, but targeted methods (multiple reaction monitoring) could be employed for that purpose and at much higher sensitivities. The latter approach appears advantageous for those proteins that are ordinarily present at low levels. Importantly, longitudinal variation in SC protein patterns obtained from the same individual at several time points will offer the opportunity to monitor response to treatment.

## Supporting Information

Figure S1 Hierarchical clustering of protein profiles. (**A**) Hierarchical clustering according to epidermal location based on pairwise comparisons. (**B**) Pairwise differences in LI (*TGM*−/−), IV and normal (Con) epidermis. (**C**) Hierarchical clustering from individuals with LI, IV, and several additional diagnoses compared to controls. AD, atopic dermatitis (nonlesional skin); Asymptomatic (Asymp), carrier of *STS* microdeletion; Compound 1 (Cmpd1), *FLG* defect with *STS* microdeletion; Compound 2 (Cmpd2), heterozygous *FLG* defect with concomitant *GJB2* mutation; normal (Con). (**D**) Samples from family I based on pairwise protein profiling. Father (F) and daughter (D) with heterozygous *FLG* defect (IV), mother (M) with normal skin. (**E**) Samples from family II, with the mother (M) being an asymptomatic *STS* microdeletion carrier, father (F) and daughter (D) with IV (heterozygous *FLG* mutation), and two sons (Son1, Son2), each heterozygous for a *FLG* mutation and exhibiting a microdeletion in *STS.*
(TIF)

Table S1 Proteins identified by analysis of epidermis from various anatomic sites. Given are the spectral counts after adjustment for shared peptides.
(XLSX)

Table S2 Results of pairwise statistical analysis of protein yields by depth using forearm samples from 3 subjects.
(XLSX)

Table S3 Results of pairwise statistical analysis of protein yields by anatomic site.
(XLSX)

Table S4 Proteins identified by analysis of epidermis from participants with various diagnoses.
(XLSX)

Table S5 Results of pairwise statistical analysis of protein yields from subjects with normal epidermis or diagnosis of lamellar ichthyosis (TGM1 negative) or ichthyosis vulgaris (FLG mutations).
(XLSX)

Table S6 Results of pairwise statistical analysis of protein yields from subjects with normal epidermis or various other diagnoses.
(XLSX)

Table S7 Results of pairwise statistical analysis of protein yields from Family I with 3 members (normal, IV).
(XLSX)

Table S8 Results of pairwise statistical analysis of protein yields from a family with 5 members (FLG+/− and +/− STS microdeletion).
(XLSX)
