# Supplementary figures and images for: Distinguishing Ichthyoses by Protein Profiling
Source: PLoS One. 2013 Oct 9;8(10):e75355. doi: 10.1371/journal.pone.0075355 (PMC3793978; doi:10.1371/journal.pone.0075355)

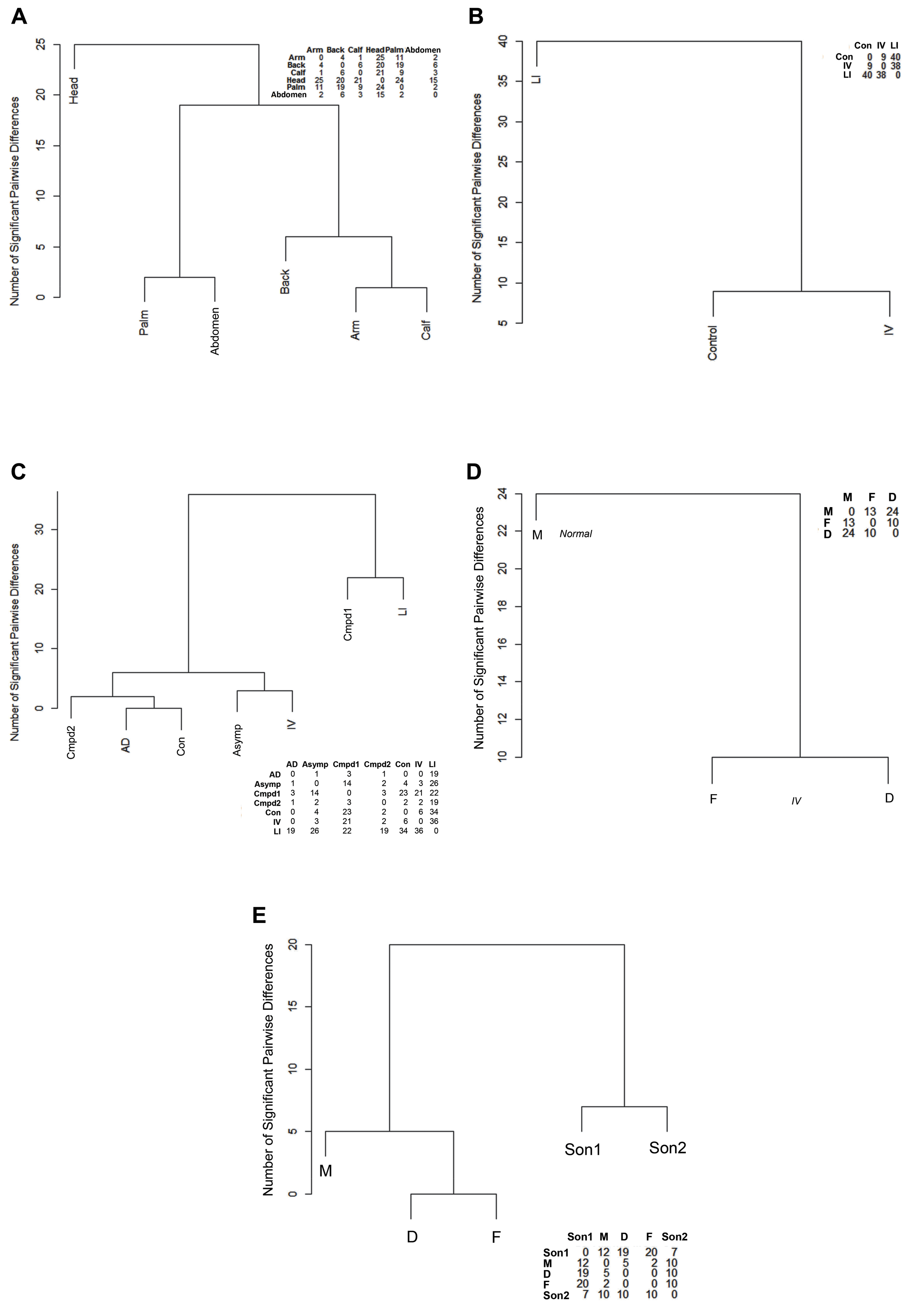

Supplement: Figure S1 — Hierarchical clustering of protein profiles. (A) Hierarchical clustering according to epidermal location based on pairwise comparisons. (B) Pairwise differences in LI (TGM−/−), IV and normal (Con) epidermis. (C) Hierarchical clustering from individuals with LI, IV, and several additional diagnoses compared to controls. AD, atopic dermatitis (nonlesional skin); Asymptomatic (Asymp), carrier of STS microdeletion; Compound 1 (Cmpd1), FLG defect with STS microdeletion; Compound 2 (Cmpd2), heterozygous FLG defect with concomitant GJB2 mutation; normal (Con). (D) Samples from family I based on pairwise protein profiling. Father (F) and daughter (D) with heterozygous FLG defect (IV), mother (M) with normal skin. (E) Samples from family II, with the mother (M) being an asymptomatic STS microdeletion carrier, father (F) and daughter (D) with IV (heterozygous FLG mutation), and two sons (Son1, Son2), each heterozygous for a FLG mutation and exhibiting a microdeletion in STS. (TIF) [file pone.0075355.s001.tif]
